# Think sepsis, write sepsis, code sepsis – patient characteristics associated with sepsis (under-)coding in administrative health data

**DOI:** 10.1007/s15010-025-02685-8

**Published:** 2025-12-08

**Authors:** Daniel Thomas-Rüddel, Norman Rose, Carolin Fleischmann-Struzek, Konrad Reinhart, Beate Boden, Heike Dorow, Andreas Edel, Falk A. Gonnert, Jürgen Götz, Matthias Gründling, Markus Heim, Kirill Holbeck, Ulrich Jaschinski, Christian Koch, Christian Künzer, Khanh Le Ngoc, Simone Lindau, Ngoc B. Mehlmann, Patrick Meybohm, Holger Neb, Michael Nordine, Dominique Ouart, Christian Putensen, Michael Sander, Jens-Christian Schewe, Peter Schlattmann, Götz Schmidt, Gerhard Schneider, Claudia Spies, Ferdinand Steinsberger, Christopher Tam, Kai Zacharowski, Sebastian Zinn, Daniel Schwarzkopf

**Affiliations:** 1 Department of Anesthesiology and Intensive Care Medicine, Jena University Hospital, Am Klinikum 1, 07747 Jena, Germany; 2 Department of Anesthesiology, Montefiore Medical Center, Albert Einstein College of Medicine, Bronx, NY USA; 3 Institute of Infectious Diseases and Infection Control, Jena University Hospital, Erlanger Allee 103, 07747 Jena, Germany; 4 Department of Anesthesiology and Operative Intensive Care Medicine (CCM, CVK), Charité-Universitätsmedizin Berlin, corporate member of Freie Universität Berlin and Humboldt-Universität zu Berlin, Augustenburger Platz 1, 13353 Berlin, Germany; 5 Department of Internal Medicine II – Intensive Care, Klinikum Lippe GmbH, Röntgenstraße 18, 32756 Detmold, Germany; 6 Department of Anaesthesiology and Intensive Care Medicine, SRH Wald-Klinikum, Straße des Friedens 122, 07548 Gera, Germany; 7 Department of Anaesthesiology, Intensive Care Medicine, Emergency Medicine and Pain Medicine, University Medicine Greifswald, Ferdinand-Sauerbruch-Straße, 17475 Greifswald, Germany; 8 TUM School of Medicine and Health, Department of Anesthesiologie and Intensive Care Medicine, Technical University of Munich, TUM University Hospital, Munich, Germany; 9 Department of Anaesthesiology and Surgical Intensive Care Medicine, Universitätsklinikum Augsburg, Stenglinstr. 2, 86156 Augsburg, Germany; 10 Department of Anesthesiology, Intensive Care Medicine and Pain Therapy, University Hospital Gießen, UKGM, Justus-Liebig University Gießen, Rudolf-Buchheim-Straße 7, 35392 Gießen, Germany; 11 Visiting Professor for Sepsis Awareness and Advocacy, Department of Anesthesiology and Operative Intensive Care Medicine (CCM, CVK), Charité-Universitätsmedizin Berlin, corporate member of Freie Universität Berlin and Humboldt-Universität zu Berlin, Augustenburger Platz 1, 13353 Berlin, Germany; 12 Faculty of Medicine, Department of Anesthesiology and Intensive Care Medicine, University of Cologne, University Hospital Cologne, Kerpener Str. 62, 50937 Cologne, Germany; 13 Department of Anaesthesiology, Intensive Care Medicine & Pain Therapy, University Hospital Frankfurt, Goethe University, Theodor- Stern-Kai 7, 60590 Frankfurt am Main, Germany; 14 Department of Anaesthesiology, Intensive Care, Emergency and Pain Medicine, University Hospital Würzburg, Oberduerrbacher Straße 6, 97080 Würzburg, Germany; 15 Department of Anaesthesiology and Intensive Care Medicine, University Hospital Bonn, Venusberg-Campus 1, 53127 Bonn, Germany; 16 Department of Anaesthesiology, Intensive Care Medicine, Emergency Medicine and Pain Medicine, University Medical Centre Rostock, Schillingallee 35, 18057 Rostock, Germany; 17 Institute for Medical Statistics, Computer Science and Data Science, Jena University Hospital, Bachstraße 18, 07743 Jena, Germany

**Keywords:** Sepsis, Epidemiology, Quality assurance, health care, Sensitivity and specificity, Administrative claims, healthcare, Hospital records

## Abstract

**Purpose:**

Sepsis is a leading cause of morbidity and mortality, yet its documentation and coding in administrative health data remain unreliable. Accurate coding is essential for epidemiological surveillance, quality assurance, and reimbursement. This study aims to identify patient characteristics associated with under-diagnosis and under-coding of sepsis in German inpatient administrative health data (IAHD).

**Methods:**

This secondary analysis of the multicenter OPTIMISE study included 10,334 hospital cases from ten German hospitals (2015–2017). Sepsis cases were identified via structured chart review and compared to ICD-coded diagnoses. Logistic regression and classification tree analyses were used to determine predictors of under-diagnosis and under-coding, including ICU admission, organ dysfunction, and infection source.

**Results:**

Among 1,310 cases fulfilling severe sepsis-1 criteria, only 30.7% were correctly coded. The strongest predictor for coding accuracy was explicit mention of sepsis in the medical chart (OR 19.58). ICU treatment, organ dysfunction severity, and mechanical ventilation were also associated with higher coding rates, while pneumonia as the infection source was linked to a lower probability of sepsis being named and coded.

**Conclusion:**

Sepsis coding in administrative data is frequently inaccurate. Explicit naming of sepsis and severity markers strongly influence correct coding. As Germany introduces mandatory sepsis quality assurance in 2026, targeted interventions – including enhanced clinician documentation and electronic coding support – are essential to improve coding reliability and patient care.

## Introduction

Sepsis, a life-threatening condition caused by the body’s response to infection [1], remains a major challenge in healthcare due to its high morbidity and mortality rates. Accurate identification and coding of sepsis cases are crucial for epidemiological surveillance, quality assurance, and optimizing patient outcomes. Correct sepsis coding in administrative health data (AHD) plays a pivotal role in hospital reimbursement, resource allocation, policy-making, and quality management. However, the reliability of sepsis coding in AHD is often low, given the potential for under-coding or misclassification, which can lead to significant biases in sepsis surveillance [2–5]. While there is a secular trend towards increased sepsis coding previous efforts to enhance coding have been of limited effect [6–8].

In a multicenter validation study of sepsis coding in German inpatient administrative health data (IAHD) we found that only one third of clinical sepsis cases were ICD-coded and that only half of the sepsis cases had been explicitly named as “sepsis” in the charts [3]. The reasons for such shortcomings in diagnosis and coding have not been studied extensively so far, with only one previous study analyzing predictors of under-coding of sepsis [9]. Understanding these factors would be essential for improving coding practices, enhancing the quality of sepsis surveillance, and ultimately, bettering patient care and outcomes. This is particularly relevant in light of the fact that a mandatory quality assurance procedure based on coding in IAHD for sepsis care will be introduced in Germany in 2026 [10]. We therefore aim to analyze the characteristics associated with under-diagnosis and under-coding of sepsis.

## Materials and methods

### Study design and setting

This manuscript presents secondary analyses based on data from the multicenter, retrospective, observational OPTIMISE study [3, 11]. The original study evaluated the validity of sepsis coding in IAHD with clinical sepsis diagnoses obtained via structured chart review. Results on the precision of sepsis coding (e.g. sensitivity, specificity) in comparison to the reference standard diagnosis, as well as results on the proportion of correctly naming sepsis in the charts have been published [3]. We now report on additional analyses conducted to identify predictors of misclassification by regressions and decision trees. Methodological details of the original study have been reported elsewhere [3, 11], and are not reiterated in full here. The study is reported in accordance with the RECORD [12] and adapted STARD guidelines for administrative data research [13].

### Sample

The study utilized data from ten hospitals across Germany, recruited through existing sepsis research and quality improvement networks. These included a mix of university and tertiary teaching hospitals. A stratified sample of hospital episodes from patients aged 15 years and older, treated between 2015 and 2017, was drawn from each site. Given the low prevalence of sepsis in hospital cases, we used a disproportional stratified sampling approach to increase the proportion of true sepsis cases. Strata were defined by the combination of ICU procedure code (*Operationen- und Prozedurenschlüssel* 8-890: yes vs. no), length of stay (≤ 6 vs. >6 days), and year of discharge (2015–2017), and 100 cases were sampled from each of the 12 strata within every hospital. The aim of the study was to review at least 1000 of the 1200 sampled episodes per hospital in random order. Full methodological details, including sampling and representativeness strategies, have been described elsewhere [3].

### Chart review

Clinical data were abstracted by trained study physicians from patient charts between July 2019 and October 2021. Prior to data collection, interrater reliability was established. Data were entered into an electronic case report form (eCRF) via OpenClinica (version 3.1. Copyright © OpenClinica LLC and collaborators, Waltham, MA, USA, www.OpenClinica.com). Reviewers were blinded to sepsis coding in the IAHD but could not be blinded to codes present in the medical documentation. The chart review data were subsequently linked to IAHD using pseudonymized identifiers. Further details have been previously reported [3].

### Variables

#### Variables derived from chart review

The eCRF was informed by prior literature and piloting [2, 4, 14]. The complete CRF has been presented in the study protocol [11]. Sepsis was assessed by study physicians based on both sepsis-1 and sepsis-3 definitions [1, 15, 16]. Since sepsis-1 criteria were in use for coding during the complete study period, analyses focus on severe sepsis-1 cases, which also align closely with clinical sepsis-3 presentations [17]. For patients with sepsis, it was documented, if “sepsis” had been named in the chart or discharge letter and trained study nurses recorded further clinical characteristics. The explicit naming of sepsis is an indicator for the adequacy of the process of recognition and clinical documentation of sepsis during the course of the treatment. Further details have been reported previously [3].

Candidate variables for investigation of associations with misclassification of sepsis were selected based on clinical reasoning: age, sex, degree of confirmation of infection, origin of infection, focus of infection, number and type of SIRS-criteria, presence of organ dysfunctions, presence of shock, treatment on ICU (yes vs. no), number and type of organ support measures and duration of mechanical ventilation (< 24 h vs. ≥ 24 h).

#### Variables derived from administrative health data

The study is based on IAHD, which are used for the reimbursement of hospitals in the German diagnosis-related groups (DRG) system. Accordingly, the ICD codes recorded in these data reflect a retrospective process that takes into account the clinical documentation as well as complex reimbursement rules and is frequently supported by specialized software. Severe sepsis-1 cases (including septic shock-1) were identified via explicit ICD-10 codes valid during the study period (R65.1 and R57.2). In addition, we defined all sepsis-1 cases by including every explicit sepsis code (e.g. A40.- and A41.-; see the supplement of the previous publication for details [3]).

### Statistical methods

Cases with a reference standard diagnosis based on chart review of severe sepsis-1 or septic shock-1 were included in the analysis. The aim of our analysis was to explore and describe typical patterns of under-diagnosis and under-coding. Therefore, we investigated the relation of candidate variables for misclassification for two classification problems: (a) naming of sepsis (true positives) vs. non-naming of sepsis (false negatives) in the chart; (b) coding of severe sepsis-1 (true positives) vs. non-coding of severe sepsis-1 (false negatives). Any mention of sepsis in the chart was considered, regardless of whether severe sepsis was explicitly named. For false negative coding of severe sepsis-1, we calculated the frequency of occurrence of any other sepsis code. For classification problem b) the naming of sepsis was itself a predictor. We did descriptive comparison between the defined groups. To assess significance of individual predictor variables, we calculated simple logistic regression analyses of the two classifications on each individual predictor and report odds-ratios (OR) with their 95% confidence intervals (CI). To identify and describe typical constellations of predictors and to incorporate complex interactions we then calculated classification trees. The minimal leaf size (i.e., the minimal number of cases in the terminal nodes) was set to 2.5% of the sample size. We used pruning to avoid overfitting of the trees to the data based on the optimal complexity parameter found empirically by means of cross-validation. Analyses were conducted using the statistical software *R* [18]. The R-package *survey* was used to calculate descriptive statistics and logistic regressions for complex data [19], which addresses sampling weights as well as the clustering within hospitals. Classification trees were calculated using the R-package *rpart*, which also allows to take sampling weights into account [20], but cannot adjust for the clustering. Missing values due to lacking information in medical records were treated by missing-data adjusted sampling weights to prevent bias by over- or underrepresentation of strata [21]. Significance tests were conducted at a bidirectional alpha level of 0.05.

## Results

Characteristics of the study sample have previously been reported [3]: A total of 10,334 charts were reviewed. Severe sepsis-1 criteria were fulfilled by 1310 cases in the sample, corresponding to 3.3% [95% CI: 2.6%, 4.1%] of cases in the full unweighted population (adjusted for sampling weights). The cases showed a mean age of 66.5 years, 40.8% were female, 65.8% had been treated on the ICU, and 50.7% had a septic shock-1.

Table 1 presents new descriptive statistics comparing false negatives with true positives for the naming of sepsis and the coding of sepsis. Only 29% of non-coded sepsis cases had been named as sepsis in the chart, while this was true for 88.9% of coded sepsis cases. Among false negatives regarding coding of severe sepsis-1 (R65.1 or R57.2), only 14.4% had received another explicit sepsis code (e.g. A40.-).

**Table 1:** Characteristics of cases with severe sepsis-1 according to the chart review

Variable	*N* of missings	All cases	Naming of sepsis in the chart		Coding of severe sepsis-1 in IAHD	
			Not named (false negatives)	Named (true positives)	Not coded (false negatives)	Coded (true positives)
Sex: female	0	40.8%	42.2%	39.3%	43.4%	35.9%
Age (years)	0	68.5 (14.8)	68.5 (15.4)	68.5 (14.2)	68.7 (15.2)	68.3 (14.1)
Degree of confirmation of infection	0					
microbiologically proven		55.8%	47.8%	64%	51.7%	63.6%
clinically suspected		17.7%	17.9%	17.5%	16.1%	20.7%
other confirmation		26.5%	34.3%	18.6%	32.2%	15.6%
Origin of infection	4					
present on admission, nosocomial		11.9%	9.1%	14.6%	12.1%	11.4%
present on admission, not nosocomial		46.3%	44.6%	48.1%	45.8%	47.4%
present on admission, unknown origin		8.4%	9.3%	7.6%	8.7%	8%
onset during stay, nosocomial		23%	24.8%	21.3%	22.8%	23.5%
onset during stay, not nosocomial		4.4%	4.7%	4.1%	4.2%	4.9%
onset during stay, unknown origin		2.3%	3.3%	1.2%	2.6%	1.6%
more than one infection		3.6%	4.2%	3%	3.8%	3.3%
Focus: catheter-related	79	4.2%	2.3%	6.2%	3.1%	6.5%
Focus: central nervous system	79	2.2%	2.7%	1.6%	2.2%	2.1%
Focus: cardiovascular	79	2%	0.8%	3.4%	1.3%	3.5%
Focus: pneumonia	79	56.7%	65.1%	48.7%	60.2%	50.8%
Focus: other upper/lower respiratory infections	79	5.6%	7.2%	4.1%	7%	3.1%
Focus: thoracic (empyema / mediastinitis)	79	1.6%	0.9%	2.3%	0.5%	3.8%
Focus: intraabdominal	79	14.6%	8.6%	21.1%	9.7%	24.1%
Focus: gastrointestinal	79	9.7%	10.8%	8.5%	10.3%	8.8%
Focus: urogenital	79	22%	16.4%	27.5%	22.5%	20.3%
Focus: bones / soft tissue	79	10%	8.3%	11.9%	10.6%	9.4%
Focus: primary bacteremia	79	8.1%	4.9%	10.2%	3.9%	14.6%
Focus: other	79	1.7%	2.1%	1.2%	1.6%	1.8%
Number of initial SIRS criteria	0	2.7 (0.7)	2.6 (0.7)	2.8 (0.75)	2.6 (0.7)	2.8 (0.8)
SIRS: tachycardia	0	67.9%	63%	72.8%	65%	73.4%
SIRS: tachypnoea	0	52.7%	50.6%	54.8%	50%	57.7%
SIRS: leukocytosis	0	80%	79.7%	80.2%	78.4%	83%
SIRS: hypothermia	0	62.4%	59.3%	65.4%	63.8%	59.6%
Number of initial organ dysfunctions	354	3.1 (1.6)	2.3 (1.3)	3.7 (1.5)	2.6 (1.4)	4 (1.4)
ODF: acute encephalopathy	246	43.6%	38.7%	48%	40.6%	48.6%
ODF: thrombocytopenia	29	29.5%	18.4%	40.7%	19.5%	49.2%
ODF: arterial hypoxemia	56	72.4%	75.6%	72.4%	71.5%	75.5%
ODF: renal dysfunction	33	40.9%	27.8%	54%	30.3%	61.1%
ODF: metabolic acidosis	46	46.4%	34.4%	59%	36.9%	64.5%
Septic shock-1	28	50.7%	36.4%	66%	40.9%	69.6%
Treated on ICU	0	65.8%	58.7%	73%	60.8%	75.4%
Number of organ support measures	48	1.6 (1)	1.4 (1)	1.9 (1)	1.4 (1)	2.1 (1)
Extracorporeal membrane oxygenation	3	2.4%	1.7%	3%	1.6%	3.9%
Renal replacement therapy	5	20.2%	10.1%	30.3%	11%	37.6%
Liver replacement therapy	1	0.1%	0%	0.2%	0.2%	0%
Vasopressor use	21	51%	39.9%	62.2%	42.4%	67.2%
Ventilation (including non-invasive)	30					
no		46.1%	51.9%	40.4%	52%	35.2%
< 24 h		9.9%	10.1%	9.5%	9.7%	10%
≥ 24 h		44.1%	38.1%	50.1%	38.2%	55%
Naming of sepsis in chart	0	49.7%			29%	89%

Descriptive statistics for *N* = 1310 cases with severe sepsis-1 according to chart review given as relative frequencies (%) or mean (SD) and calculated adjusting for the sampling design. Values are missing, if the chart did not contain enough information to obtain the data. IAHD: inpatient administrative health data. ICU: intensive care unit. SIRS: systemic inflammatory response syndrome. ODF: Organ dysfunction

### Predictors of naming sepsis

**Fig. 1:**
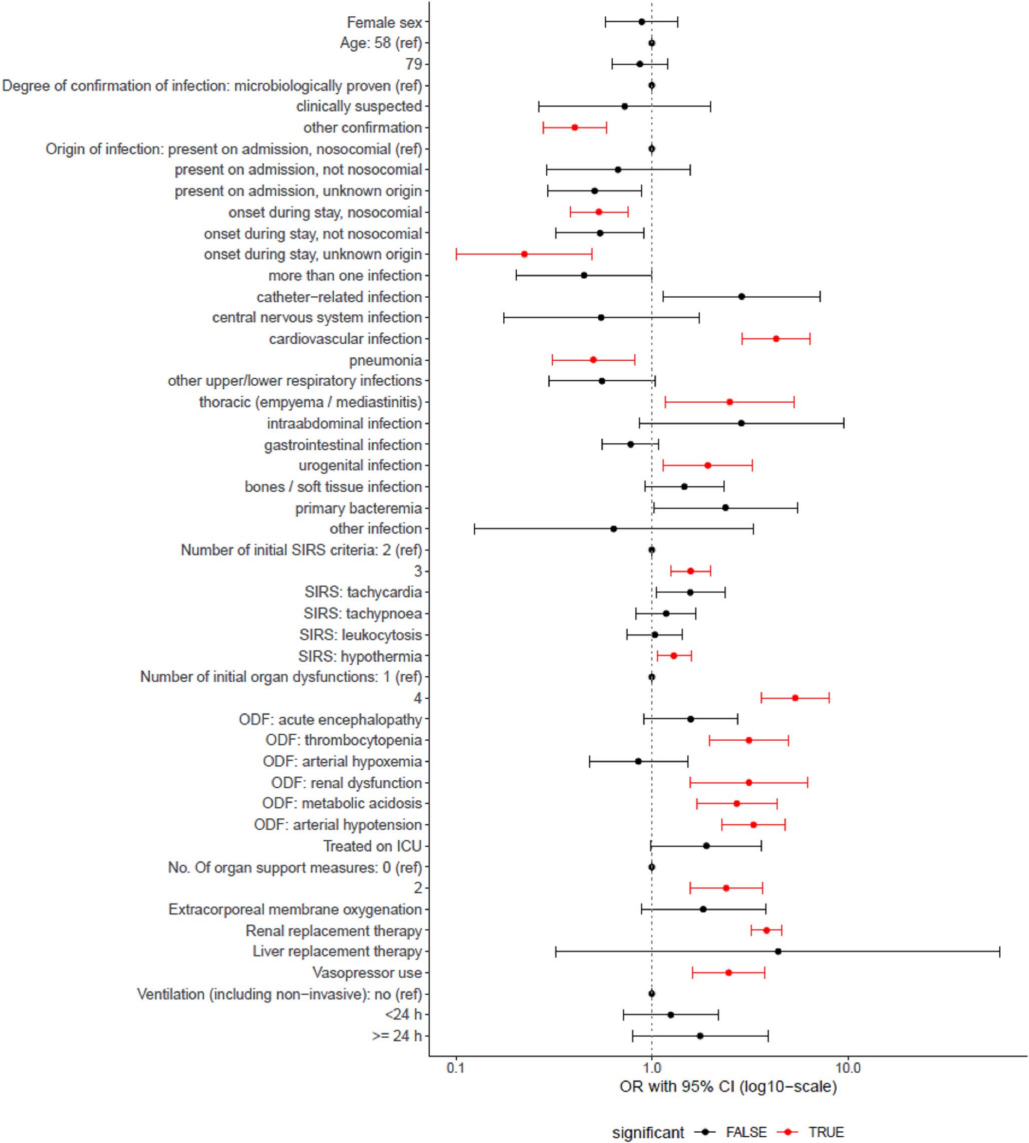
Odds ratios (log. scale) from univariate logistic regressions for the prediction of correct naming of sepsis in the charts of severe sepsis-1 cases

All the following results are presented for the whole population taking sampling weights into account. In univariate regression analysis (Fig. 1), indicators of a more severe critical illness were associated with increased odds of sepsis being named in the charts (number of SIRS criteria, number of organ dysfunctions, number of organ support measures). Characteristics of the infection also showed significant influences. While other means of infection confirmation (compared to microbiological), onset of infection during the current hospital stay, and pneumonia as source of sepsis as identified by chart review decreased the odds of naming sepsis, cardiovascular, thoracic, and urogenital foci increased the odds.

Results obtained by complex logistic regression models adjusting for the sampling design. Red color marks odds ratios (OR), which are significantly different from 1 (*p* ≤ 0.05). OR for continuous variables were obtained comparing the 1st quartile (reference) to the 3rd quartile. Statistical significance and the fact that the 95% confidence interval (CI) excludes 1 can differ slightly from each other in complex logistic regression.

**Fig. 2:**
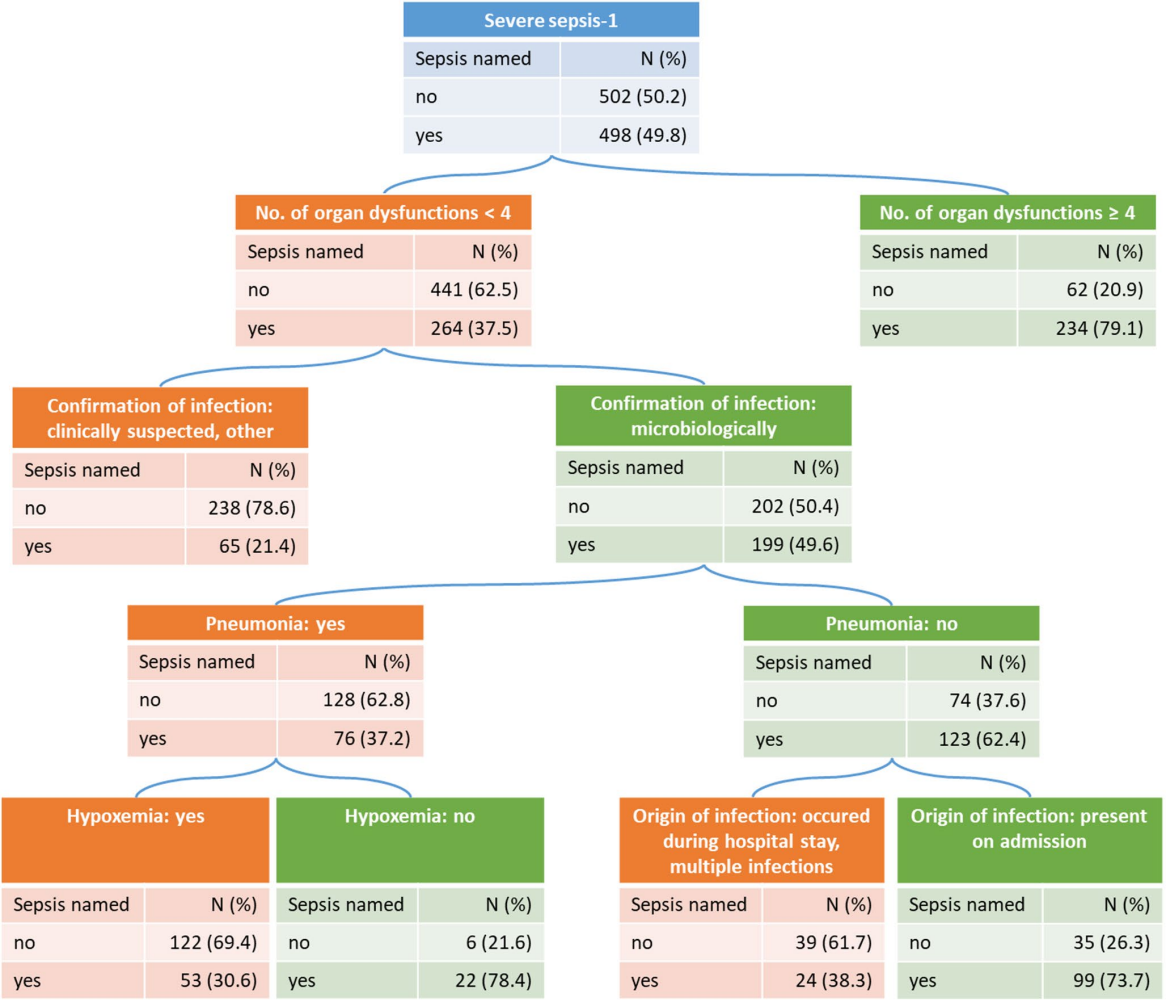
Pruned classification tree for the prediction of naming of sepsis in the patient’s charts among severe sepsis-1 cases identified by chart review, adjusted for sample weights

Results of the classification tree analysis are presented in Fig. 2. Only 49.8% of cases with severe sepsis-1 had been named as having sepsis in the chart. In total, the tree identified six terminal nodes representing distinct groups with different probabilities of being named with sepsis, showing a fair discrimination (AUC = 0.78). The strongest separation between true positives and false negatives was achieved by the number of organ dysfunctions at a cut-off of ≥ 4. Additional relevant variables were type of confirmation of infection, presence of pneumonia, presence of hypoxemia and origin of infection.

The absolute numbers presented are standardized to a sample of *N* = 1000 cases with severe sepsis-1 to improve interpretability of the chart. The tree achieved an area under the curve (AUC) of 0.78.

### Predictors of coding of severe sepsis-1

**Fig. 3:**
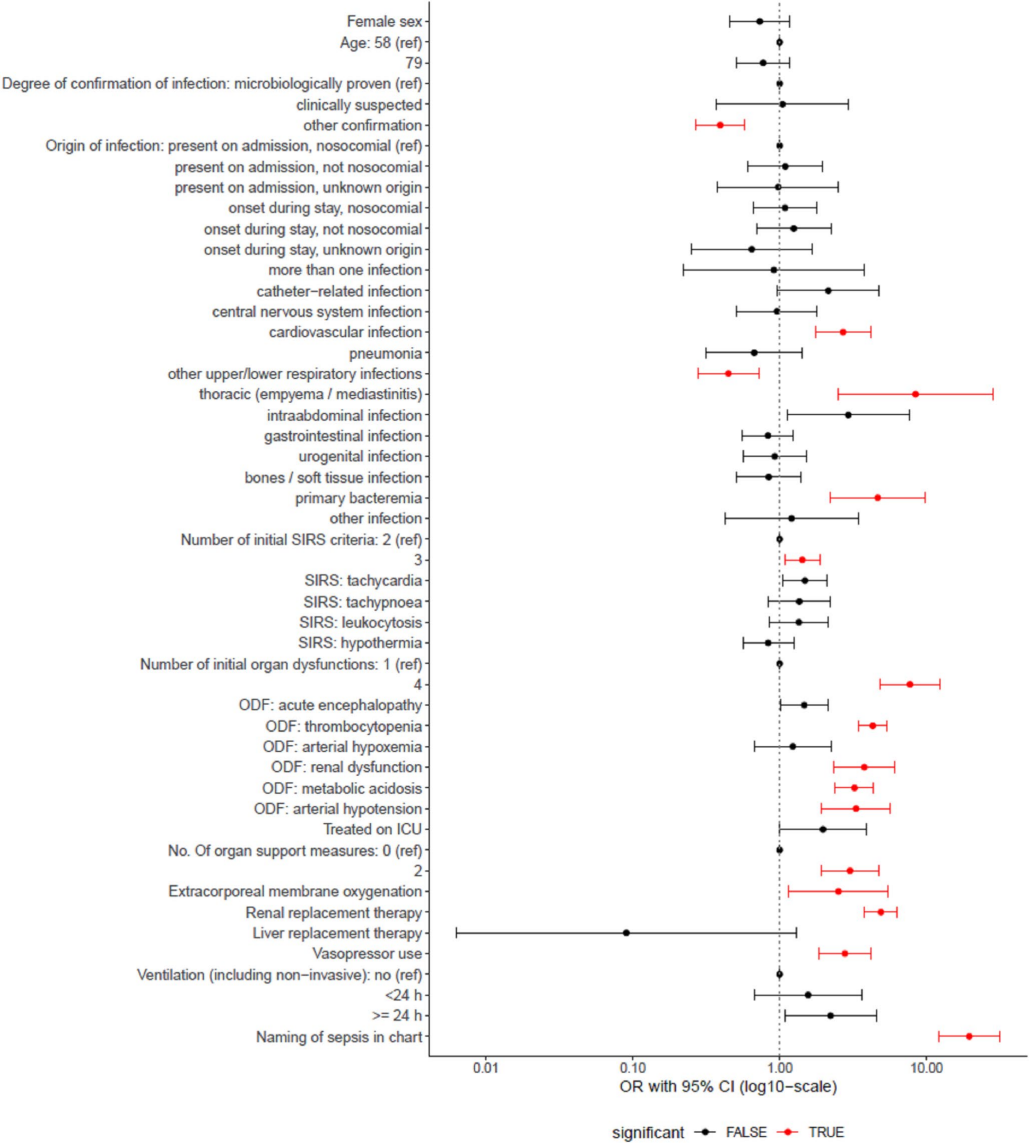
Odds ratios (log. scale) from univariate logistic regressions for the prediction of true positive coding of severe sepsis-1

In the overall sample, 30.7% of severe sepsis-1 cases were correctly coded. The strongest predictor of true positive coding of severe sepsis-1 in univariate regression was the naming of sepsis in the medical chart (OR 19.58 [12.06; 31.75], Fig. 3). Like for naming of sepsis, several indicators of a more severe critical illness increased the odds of a correct coding. While other types of confirmation of the infection and a respiratory focus of infection decreased the odds of coding, a cardiovascular focus, a thoracic focus and primary bacteremia increased the odds.

Results obtained by complex logistic regression models adjusting for the sampling design. Red color marks odds ratios (OR), which are significantly different from 1 (*p* ≤ 0.05). Statistical significance and the fact that the 95% confidence interval (CI) excludes 1 can differ slightly from each other in complex logistic regression.

**Fig. 4:**
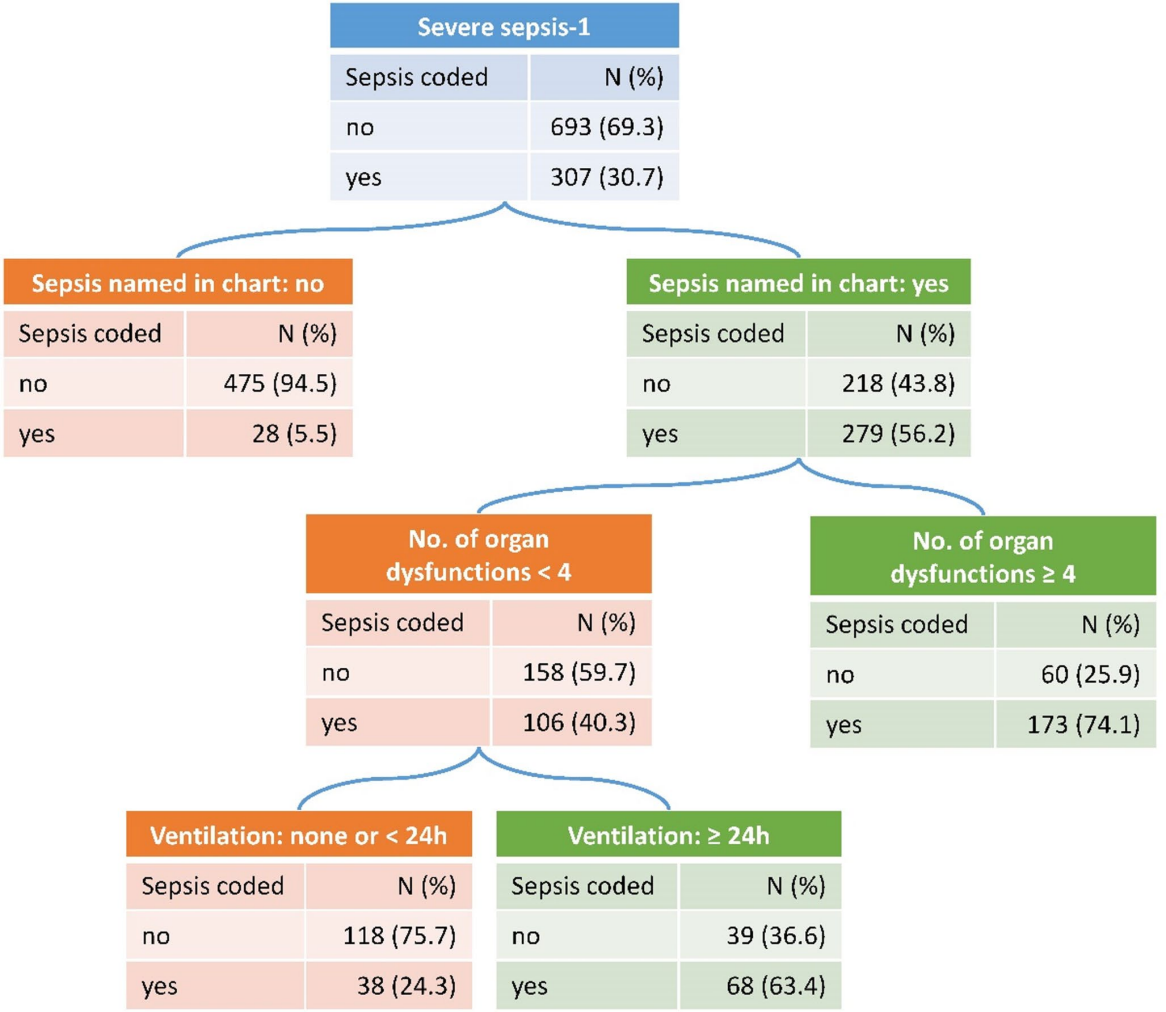
Pruned classification tree for the prediction of coding severe sepsis-1 in inpatient administrative health data among severe sepsis-1 cases identified by chart review, adjusted for sample weights

The classification tree analysis on correct coding of severe sepsis-1 identified four leaf nodes (Fig. 4), showing a good discrimination (AUC = 0.87). Naming of sepsis achieved the strongest separation between false negatives and true positives. If a case was not named as sepsis, it had a very low probability of being coded in administrative data (5.5%), but even among named sepsis cases, only 56.2% were coded. Among these, additional separation was achieved involving the variables number of organ dysfunctions and conduction of mechanical ventilation.

The absolute numbers presented are standardized to a sample of *N* = 1000 cases with severe sepsis-1 to improve interpretability of the chart. The tree achieved an area under the curve (AUC) of 0.87.

## Discussion

Sepsis coding in IAHD is often inaccurate. Extending previously reported results from our validation study [3], our further analyses found that the explicit naming of sepsis in medical charts was the strongest predictor for accurate sepsis coding. Strong predictors of sepsis naming were several measures of disease severity, ICU treatment and organ support and an extra-pulmonary focus of infection.

Our findings about organ support and ICU admission as strong predictors of sepsis coding are in line with other studies [9, 22–25]. The same has been reported previously for severity of illness assessed by APACHE II and the presence of shock [9] or APACHE II and SOFA score [25], while in other studies severity of illness was measured by mortality only [22, 23, 26]. Interestingly in one study this difference disappeared when a wider list of ICD codes, especially for pneumonia, infection exacerbated COPD and urinary tract infection, was used to identify sepsis [25].

The association of documenting sepsis in the medical chart and correct coding has been described previously in a review of 100 patients [9]. 27 cases were correctly coded as severe sepsis or septic shock and all (100%) had sepsis named in the medical record. 73 cases were wrongly not coded as sepsis and only 3 (4%) had sepsis mentioned in the medical record. In a multivariable logistic regression model this paper found that higher baseline APACHE II scores, the presence of shock at admission, higher initial serum lactate levels, bacteremia as the source of infection and admission to an ICU had unique predictive power for assigning an ICD-9 code specific for severe sepsis or septic shock [9]. Those results are all in line with our findings, while the association of pneumonia and under-coding was not seen in this study from 2013. For pneumonia an extremely high variation between hospitals regarding sepsis coding has been reported before [27, 28]. As mentioned above, adding codes for pneumonia and infection exacerbated COPD to an ICD-10 based sepsis definition increased sensitivity with only small decreases in specificity. As respiratory infections are the most frequent focus of infection in sepsis patients [29, 30], this results in a high overall rate of under-coding and changes in coding of pneumonia with organ dysfunction have great influence on overall numbers.

Looking at the overall low reliability of coding – even in cases where sepsis was named in the chart – it seems to be questionable if training and awareness alone are sufficient to improve coding practices to a sufficient degree. The fact that sepsis can occur in almost all departments of a hospital makes usual improvement strategies of training and feedback additionally challenging. Electronic support systems embedded into electronic health records might be more reliable and already show promise in sepsis diagnosis and prediction [31]. Another possible reason for under-coding of sepsis might be denial of the diagnosis by payers. In the US, sepsis was reported to be one of the top most denied codes [32]. There are no respective statistics for Germany, but this observation was repeatedly confirmed by medical controllers in our discussions. Missing documentation of clinical indicators are the most common reasons for denials [32], which might partly explain why sepsis cases with fewer organ dysfunctions were not coded as sepsis in our data. Experiencing denials and associated penalties might also incentivise a habit of under-coding among medical controllers. This complex issue needs further investigation.

Our study has several strengths. It is the first to investigate predictors of naming sepsis in the chart and the interrelation between naming and coding of sepsis, it uses a complete chart review and a multicenter design, while previous studies on validity of sepsis coding were largely monocentric [4]. As the heterogeneity between hospital is large in our cohort, but also in other studies [26, 28], a greater number of study centers might be needed to get really representative results, but single-center studies have to be interpreted with great caution. Since, to our knowledge, there are no appropriate statistical methods to incorporate pattern differences between clusters in complex samples, we cannot exclude that systematic differences in coding practices between hospitals have biased our results. Information might not be missing at random, which could also bias our results. As this study was performed in Germany it may not be fully generalizable to other countries with different national ICD-10 versions and different coding and reimbursement rules. SEPSIS-3 criteria were published in 2016 during the analyzed period but not implemented in the coding guidelines in Germany before 2020. Since the changes in coding rules may have materially altered documentation and coding behavior, our results might not be fully applicable to current practice and need replication. We conducted a retrospective analysis and can only show associations but no prediction of actual decision processes of clinicians or medical coders.

## Conclusion

Sepsis coding in administrative health data is frequently inaccurate, with explicit naming in medical charts and severity of illness being the strongest predictors for correct coding. Future efforts to improve coding should focus on cases that are most often not coded like those treated on normal wards and those with pneumonia as a focus. The German Quality Network Sepsis along with the German Sepsis Society has issued a guide to standardize and clarify rules for documentation and coding of sepsis [33]. Clinical staff needs to be trained on documentation requirements, also to prevent denials of sepsis diagnoses by payers. Likewise, coding staff needs to be aware of the clinical criteria of sepsis. Collaboration between physicians, medical coders and quality assurance professionals is essential to improve the data quality for the upcoming mandatory quality assurance procedure of sepsis care in Germany. Electronic support systems may also help to achieve adequate coding.

## Data Availability

The study protocol has been published with open access (https://bmjopen.bmj.com/content/10/10/e035763.info). Deidentified participant data are available from the corresponding author on reasonable request (E-Mail: Daniel.Schwarzkopf@med.uni-jena.de). Access to anonymised data might be granted following review and permission of a study proposal by the ethics commission and data protection officer of the Jena University Hospital, as well as by the involved study centers.
